# The evaluation of using new trachea and skin manikins for practicing emergency anterior neck access

**DOI:** 10.1186/s12245-021-00350-z

**Published:** 2021-05-01

**Authors:** Sumidtra Prathep, Wilasinee Jitpakdee, Pittayapon Pitathawatchai, Sittichoke Anuntaseree

**Affiliations:** 1 Department of Anesthesiology, Prince of Songkla University, 15 Kanjanavanich Road, Hat Yai, Songkhla, 90110 Thailand; 2 Department of Otolaryngology Head and Neck Surgery, Prince of Songkla University, Songkhla, Thailand; 3 Department of Orthopedic Surgery, Prince of Songkla University, Songkhla, Thailand

**Keywords:** Neck, Model, Airway

## Abstract

Emergency anterior neck access may be performed if intubation and ventilation fail. Practicing this life-saving procedure with manikins before facing a real-life emergency anterior neck access is required to do this procedure successfully when we encounter a difficult airway situation. The current manikins are expensive and thus are sometimes difficult to acquire in low-cost settings such as Thailand. We devise a cost-effective training manikin using less expensive materials but retaining the simple design of the trachea and skin areas which are flexible polyurethane (PU) foam and silicone, but which still had the same utility as the current manikins. Five items were evaluated, and then scores were rated by experienced physicians from 1 to 5 points for each item, 1 being the least and 5 the highest. The mean score concerning the appropriate size of the manikins was 4.55 ± 0.56. The mean score of the ease of use for practicing was 4.58 ± 0.59. The mean score of the similarity of the skin of the manikins to human skin was 3.85 ± 0.66. The mean score of the similarity of the trachea of the manikins to the human trachea was 3.80 ± 0.69. The mean score of the sensation of inserting the tube in the manikin compared to a real trachea was 3.90 ± 0.67. The mean overall benefit score of practicing on the manikins was 4.38 ± 0.45. Our trial indicates that this low-cost and simply designed manikin can be useful for practicing emergency airway management procedures to save patients who are struggling with lack of oxygen or intubation failure or failure of ventilation or other airway equipment such as endotracheal intubation and supraglottic airway devices (SGA).

## Introduction

A difficult airway is defined as the clinical situation in which a conventionally trained anesthesiologist or other specially trained clinician experiences difficulty with face mask ventilation of the upper airway and tracheal intubation. The incidence of difficult intubation in the hospital setting has been reported from 0.5 to 12.95% [1, 2] and the incidence of difficult ventilation from 0.9 to 12.8% [3, 4]. Failed intubation or ventilation can result in hypoxia and/or hypoxemia, leading to various critical situations such as hypoxic cardiac arrest [5, 6]. Emergency anterior neck access must be performed if intubation and ventilation fail and is the final step of the Difficult Airway Society guideline (DAS) [7] and Practice Guidelines for Management of the Difficult Airway by the American Society of Anesthesiologists (ASA) Task Force on Management of the Difficult Airway [8].

Anterior neck access is a race against the clock. The patient can survive if the procedure is performed in time. Practicing this life-saving procedure with manikins before facing a real-life emergency anterior neck access is required to give the physician confidence in their ability to do this procedure successfully when we encounter a difficult airway situation. The current manikins are expensive and thus are sometimes difficult to acquire in low-cost settings such as Thailand.

The aim of this study was to devise a cost-effective training manikin using less expensive materials but retaining the simple design of the trachea and skin areas, but which still had the same utility as the current manikins for the students, residents, and fellows who need to practice emergency anterior neck access before facing critical patients.

## Methods

The study was conducted in the PSU Airway Management Excellence Center (PAMEC) of Songklanagarind Hospital, an 856-bed medical center in Hat Yai, Thailand, affiliated with Prince of Songkla University (PSU), and the primary tertiary care and referral center in southern Thailand.

After approval from the Institutional Ethics Committee, the medical staffs and last year residents of the Departments of Emergency Medicine, Otolaryngology (Head and Neck Surgery), Surgery, and Anesthesiology of our institution were invited to participate in the study, which was conducted during June and July 2020, with the purpose of testing the new manikins for their usefulness in practicing emergency anterior neck access, including a realistic skin presentation. All physicians experienced practice with previous commercial manikins before. All surgeons and otolaryngology doctors performed tracheostomy on the real patients before.

## Design

Before we started to design our new special-purpose manikin, we searched for existing patents for manikins which were similar to our proposed new design from patentscope.wipo.int using the keywords of neck, model, and airway and found patent numbers US20140302475 [9], US20170345341 [10], US20140154656 [11], and CN209000347 [12] which are for manikins for practicing cricothyroidotomies. However, the DAS guideline 2015 suggests changing to a large bore tube, such as endotracheal tube No.6.0 as we performed in this trial.

Due to the high cost of the manikins available for practice currently, which make them difficult to obtain in many developing countries such as Thailand, we decided to try to design a new manikin which would have a lower production cost yet the same lifelike features of the available manikins which would allow us to offer practices of this procedure to more people affordably.

**Fig. 1:**
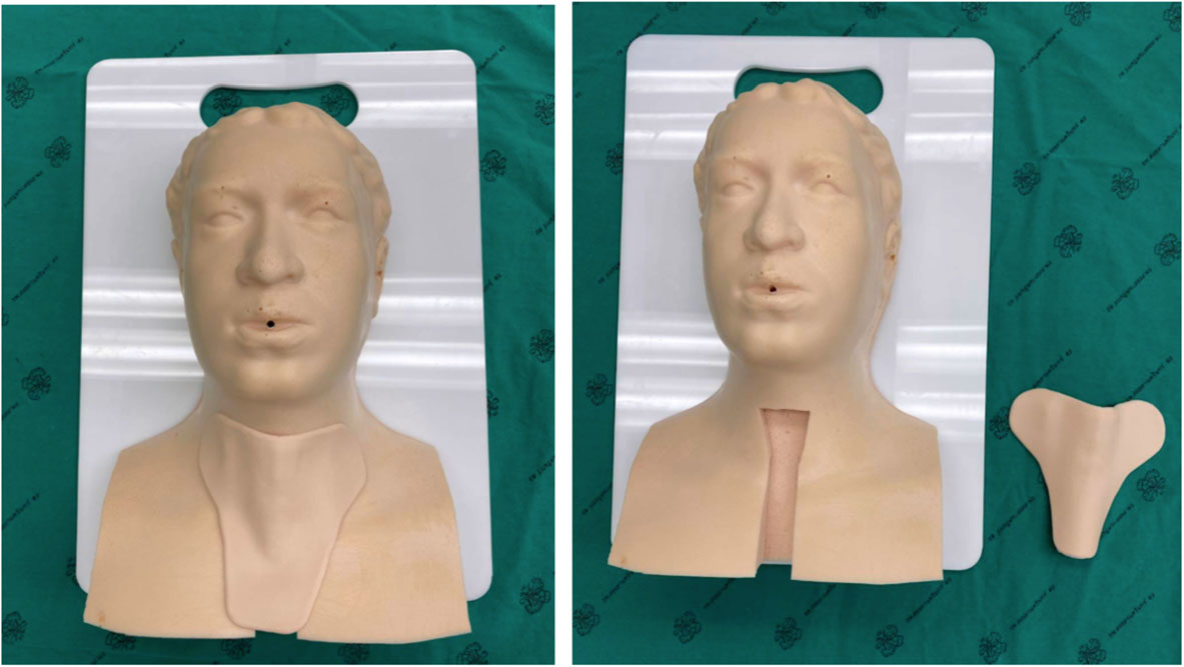
The two parts of the manikin; the body and trachea/skin covering

**Fig. 2:**
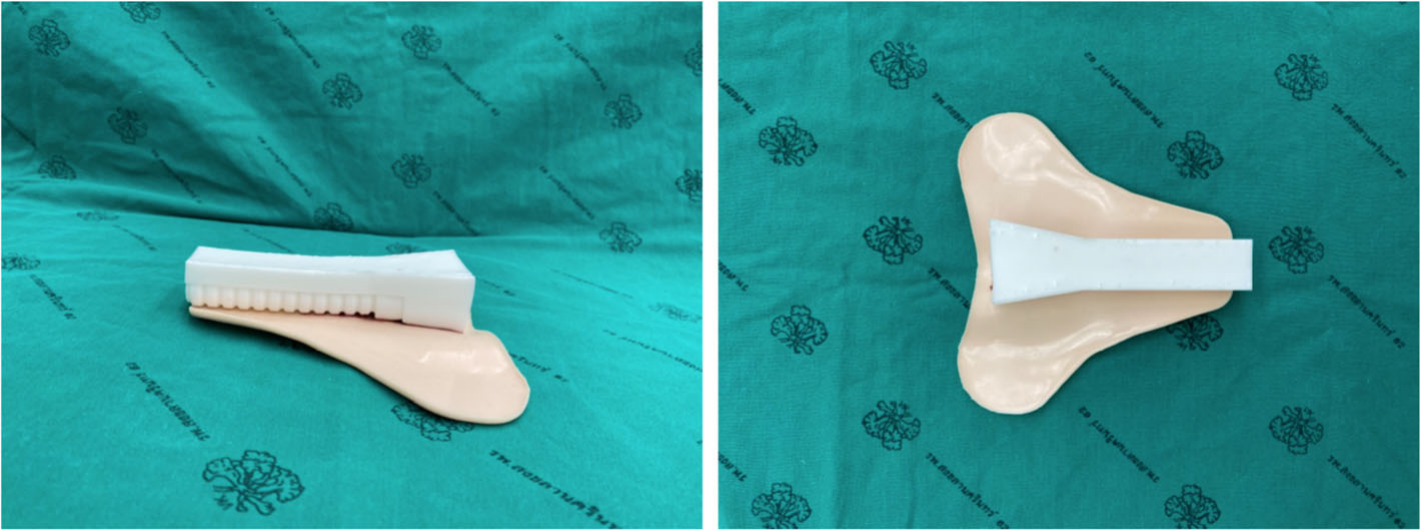
Lateral and posterior views of the trachea and skin covering

We designed a prototype manikin consisting of two parts. First was the body, made from flexible polyurethane (PU) foam as a light and durable material (Fig. 1). The molded fiberglass was shaped as we designed as the actual size of head and neck. There was a space to put the tracheal model on it then we mixed the polyether polyol and isocyanate to be PU foam model. Second, silicone, a material which has the property of elasticity similar to the trachea and surrounding skin (Fig. 2) to create a trachea-surrounding skin section after the molded fiberglass, was designed as the actual size. Both silicone and PU foam are inexpensive, and thus manikins can be created from these materials inexpensively for distribution to medical training centers with high cost-effectiveness.

We created a prototype manikin and did some kind of pre-trial evaluation before testing by experienced physicians to assess the feasibility of using the new manikins for practice.

After the materials testing, we gathered a group of 40 experienced airway management physicians and last year residents from the Departments of Emergency Medicine, Otolaryngology (Head and Neck Surgery), Surgery, and Anesthesiology and asked them to evaluate their level of satisfaction with various aspects of practicing emergency anterior neck access with the new manikins.

Five items were evaluated, the size of the manikins, the ease of use, the similarity of the skin and trachea of the manikins compared with the actual human frontal neck area, the sensation of inserting the tube, and the overall benefit of the practice with the manikins. The satisfaction scores were rated from 1 to 5 points for each item, 1 being the least satisfaction and 5 the highest satisfaction.

## Results

**Fig. 3:**
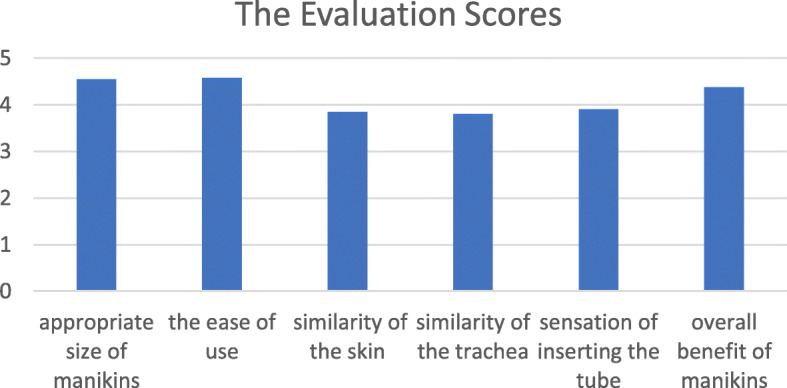
The evaluation scores of the usefulness of the new manikin for practicing emergency anterior neck access

The mean score concerning the appropriate size of the manikins was 4.55 ± 0.56. The mean score of the ease of use for practicing was 4.58 ± 0.59. The mean score of the similarity of the skin of the manikins to human skin was 3.85 ± 0.66. The mean score of the similarity of the trachea of the manikins to the human trachea was 3.80 ± 0.69. The mean score of the sensation of inserting the tube in the manikin compared to a real trachea was 3.90 ± 0.67. The mean overall benefit score of practicing on the manikins was 4.38 ± 0.45 (Fig. 3).

There were many comments and suggestions offered by the participants, such as these manikins were useful for the physicians to practice the technique, and the resistance of the silicone skin was similar to human skin. Suggestions were also made about improving the usefulness of the manikins, such as making the cricoid cartilage, thyroid cartilage, and trachea harder and adding different layers of subcutaneous tissue and a thyroid notch to the manikins which we plan to use in a modified version of the manikin.

## Discussion

Difficult airway is one of the health issues requiring advanced airway training including members from the Department of Emergency Medicine, Otolaryngology (Head and Neck Surgery), Surgery, Internal Medicine, and Anesthesiology. Our hospital, Songklanagarind Hospital, affiliated with Prince of Songkla University (PSU), opened the PSU Airway Management Excellence Center (PAMEC) in 2015 to provide specialized training and services for medical personnel required to be prepared for emergency airway management situations.

The standard guidelines for difficult airway ventilation and intubation we follow are the Difficult Airway Society guideline (DAS) [7] from Europe and the Practice Guidelines for Management of the Difficult Airway of the American Society of Anesthesiologists (ASA) Task Force on Management of the Difficult Airway [8] from the USA. Both guidelines are similar, recommending emergency anterior neck access if intubation and ventilation fail.

Emergency anterior neck access is a life-saving procedure if it can be performed in time to avoid critical desaturation and hypoxic cardiac arrest. Since this is a rare procedure, the physician must practice with manikins before facing a real difficult airway patient to acquire and maintain the ability to perform it in a true emergency situation.

We found similar manikins with US patent numbers US20140302475 [9], US20170345341 [10], US20140154656 [11], and CN209000347 [12] for manikins which are used for practicing cricothyroidotomies. These manikins include a mandible and neck base structure with removable and replaceable larynxes and are quite expensive. In place of the high-cost parts, we designed simple and low-cost structures from silicone and flexible PU foam including a removable trachea and skin structure. Overall cost is approximately 40% lower than the cost of commercial manikins in the market.

In this study, experienced physicians from the Departments of Emergency Medicine, Otolaryngology (Head and Neck Surgery), Surgery, Internal Medicine, and Anesthesiology tested these manikins and evaluated their satisfaction with the usefulness of the manikins for practicing emergency anterior neck access in cases of difficult airway. The participants indicated overall high levels of satisfaction in the six dimensions we evaluated, the appropriate size of the manikins, the ease of use, the similarity of the skin and trachea compared with human skin, the sensation of inserting the tube compared to a real human, and the overall benefit of using the manikins to practice emergency anterior neck access, with a final overall rating that the new manikins were very acceptable for practicing this technique.

Our trial indicates that this low-cost and simply designed manikin can be useful for practicing emergency airway management procedures to save patients who are struggling with lack of oxygen or intubation failure or failure of ventilation. In such situations, an immediate life-saving procedure is needed to save the patient by experienced physicians, who gain experience in such procedures by practicing with manikins.

## Conclusions

Emergency anterior neck access must be performed if intubation and ventilation fail, and is the final step of the Difficult Airway Society guideline (DAS) and Practice Guidelines for Management of the Difficult Airway by the American Society of Anesthesiologists (ASA) Task Force on Management of the Difficult Airway. Practicing these manikins before facing a real-life emergency anterior neck access is required to give the physician confidence in their ability to do this procedure successfully when we encounter a difficult airway situation. This study shows a cost-effective training manikin using less expensive materials but retaining the simple design of the trachea and skin areas which are flexible polyurethane (PU) foam and silicone, but which still had the same utility as the current manikins. Our trial indicates that this low-cost and simply designed manikin can be useful for practicing emergency airway management procedures.

## Data Availability

The data supporting the findings of this study are available within the article. We have not used the name and surname of the assessors which could not access to the data except the authors.

## Abbreviations

DAS: Difficult Airway Society guideline
ASA: American Society of Anesthesiologists
PSU: Prince of Songkla University
PAMEC: Airway Management Excellence Center
PU: Polyurethane
